# Mixed adenoneuroendocrine carcinoma of the esophagogastric junction: a case report

**DOI:** 10.1186/s40792-018-0464-x

**Published:** 2018-06-14

**Authors:** Miho Yamamoto, Soji Ozawa, Kazuo Koyanagi, Junya Oguma, Akihito Kazuno, Yamato Ninomiya, Kentaro Yatabe, Kazuhito Hatanaka

**Affiliations:** 1 Department of Gastroenterological Surgery, Tokai University School of Medicine, 143 Shimokasuya, Isehara, Kanagawa 259-1193 Japan; 2 Department of Pathology, Tokai University School of Medicine, 143 Shimokasuya, Isehara, Kanagawa 259-1193 Japan

**Keywords:** Mixed adenoneuroendocrine carcinoma, Esophagogastric junction

## Abstract

**Background:**

Mixed adenoneuroendocrine carcinoma (MANEC) is a tumor of the gastrointestinal tract that contains both exocrine and endocrine components, with each component exceeding 30% of the total tumor area. Because MANECs are exceedingly rare, no therapeutic strategies have been established yet.

**Case presentation:**

An 81-year-old man was referred to our hospital with a 5-month history of dysphagia. Esophagogastroduodenoscopy revealed an ulcerated mass in the lower thoracic esophagus, extending up to the esophagogastric junction (33 to 40 cm from the incisors). The initial biopsy diagnosis was adenocarcinoma. Computed tomography revealed no evidence of lymph node or distant metastasis. The patient was treated by thoracoscopic esophagectomy with three-field lymph node dissection and gastric tube reconstruction via a posterior mediastinal approach, under the diagnosis of esophagogastric junctional cancer (T3N0M0, stage IIA). Histopathological examination revealed two distinct components, namely, a neuroendocrine carcinoma component and an adenocarcinoma component, and the patient was diagnosed as having mixed adenoneuroendocrine carcinoma (MANEC). He presented with liver metastasis 6 months after the surgery. Thereafter, the tumor became even more aggressive, and the patient died 8 months after the surgery.

**Conclusions:**

We report a patient with MANEC of the esophagogastric junction. Close attention should be paid to such patients, as MANEC can be a highly aggressive tumor, showing rapid progression. In the treatment of MANEC, it is necessary to carefully consider the pathological features in each individual case.

## Background

Mixed adenoneuroendocrine carcinoma (MANEC) is a rare tumor of the gastrointestinal tract that contains both exocrine and endocrine components, with each component exceeding 30% of the total tumor area [1]. In this report, we describe a patient with MANEC of the esophagogastric junction.

## Case presentation

**Fig. 1:**
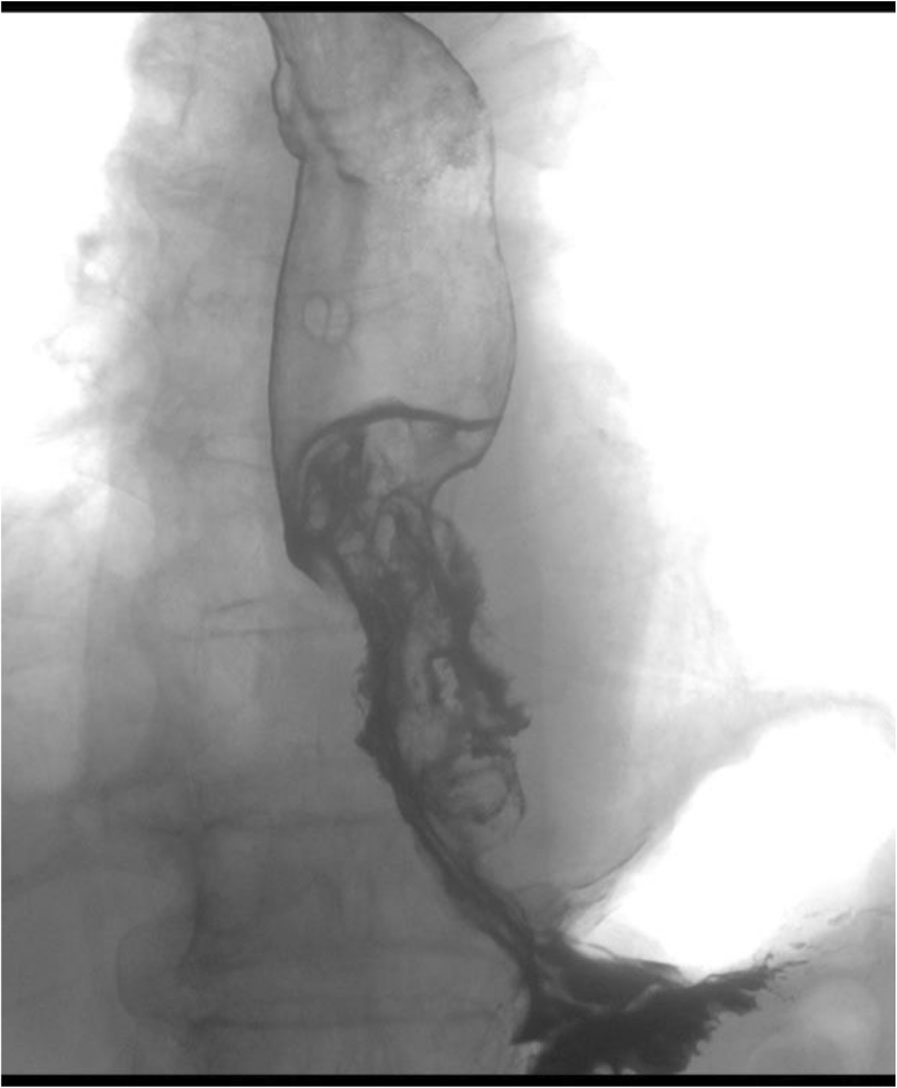
A barium esophagogram revealed an 85-mm long mass with ulceration

**Fig. 2:**
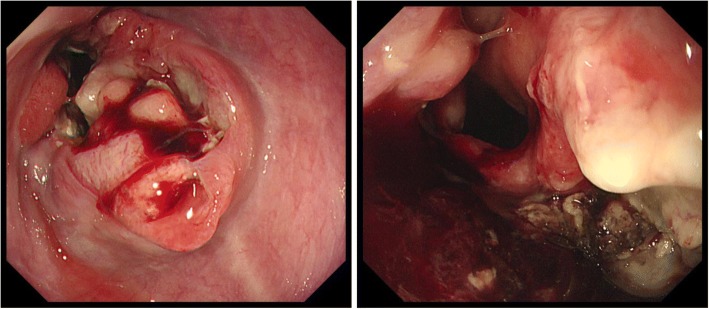
Esophagogastroduodenoscopy (EGD) revealed an ulcerated mass, 33 to 40 cm from the incisors

**Fig. 3:**
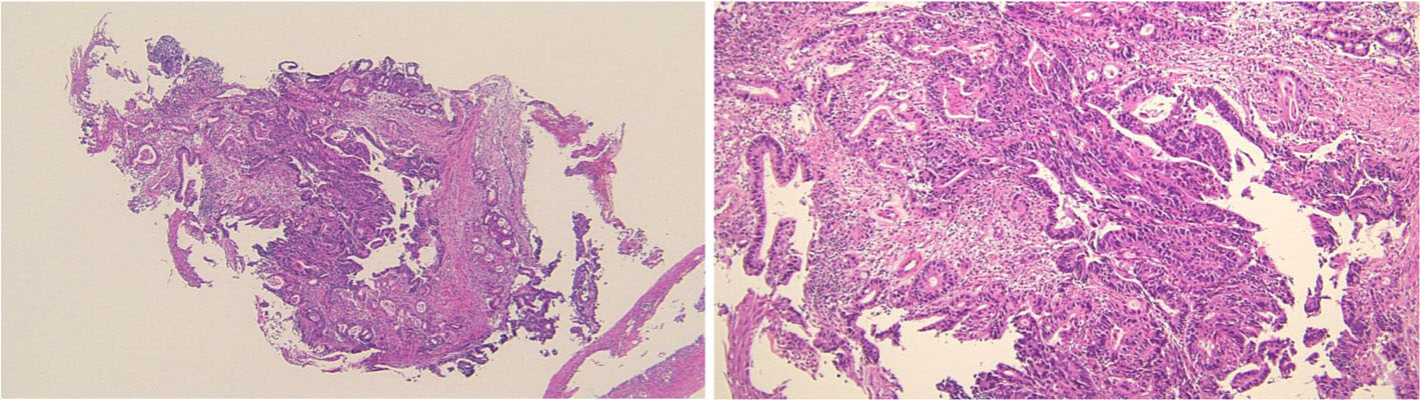
Histopathological examination of an endoscopic biopsy specimen showed proliferation of atypical cells with glandular structures, based on which the tumor was diagnosed as an adenocarcinoma

**Fig. 4:**
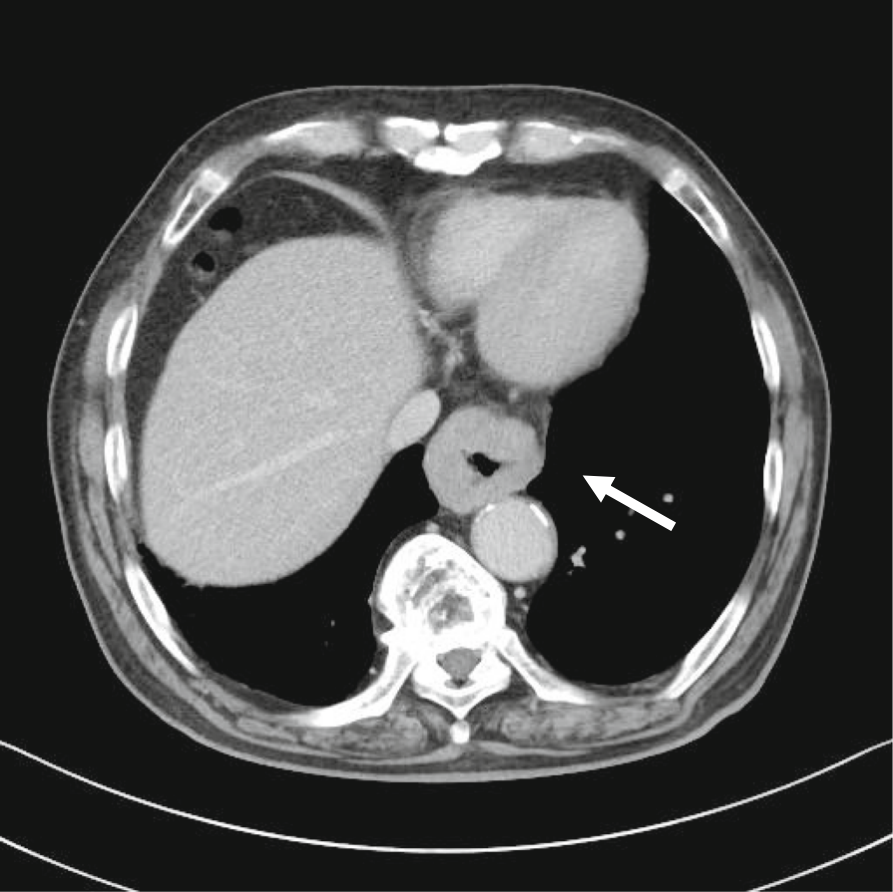
Computed tomography (CT) of the thorax revealed wall thickening from the lower thoracic esophagus to the abdominal esophagus (arrow)

**Fig. 5:**
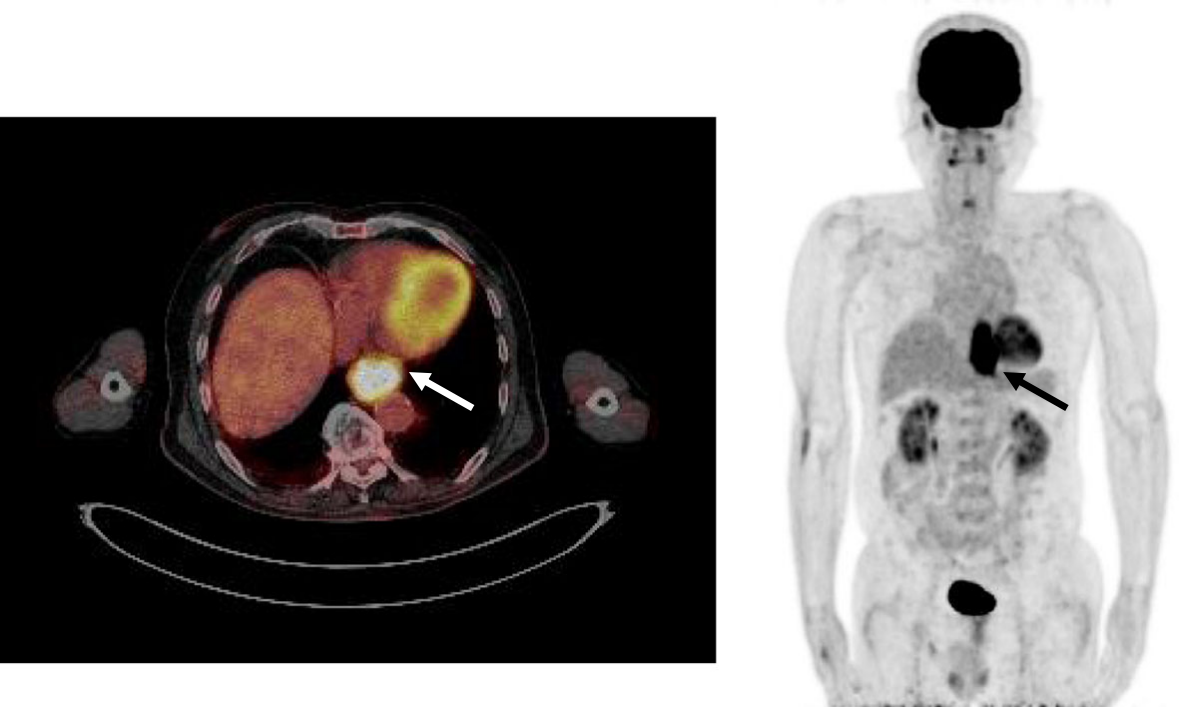
Positron emission tomography (PET)-CT revealed accumulation of fluorine-18-2-fluoro-2-deoxy-d-glucose (FDG) in the lower thoracic esophagus, with a standardized uptake value of 8.8 (arrow)

**Fig. 6:**
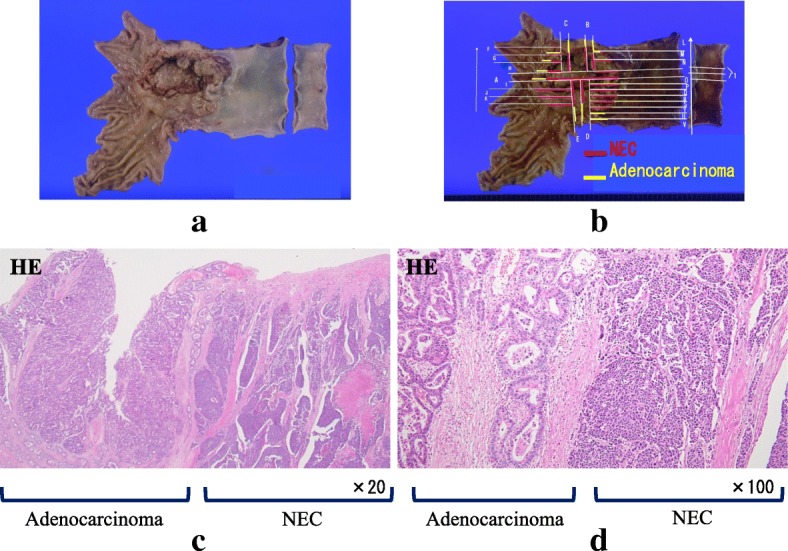
Surgical specimen and histopathological examination. **a**, **b** Surgical specimen of the esophagogastric junction. An ulcerated mass measuring 10.0 × 7.5 cm in size was seen. **c**, **d** Histopathological examination of the resected specimen revealed two distinct components, an adenocarcinoma component and a NEC component, with no zone of transition between the two components

**Fig. 7:**
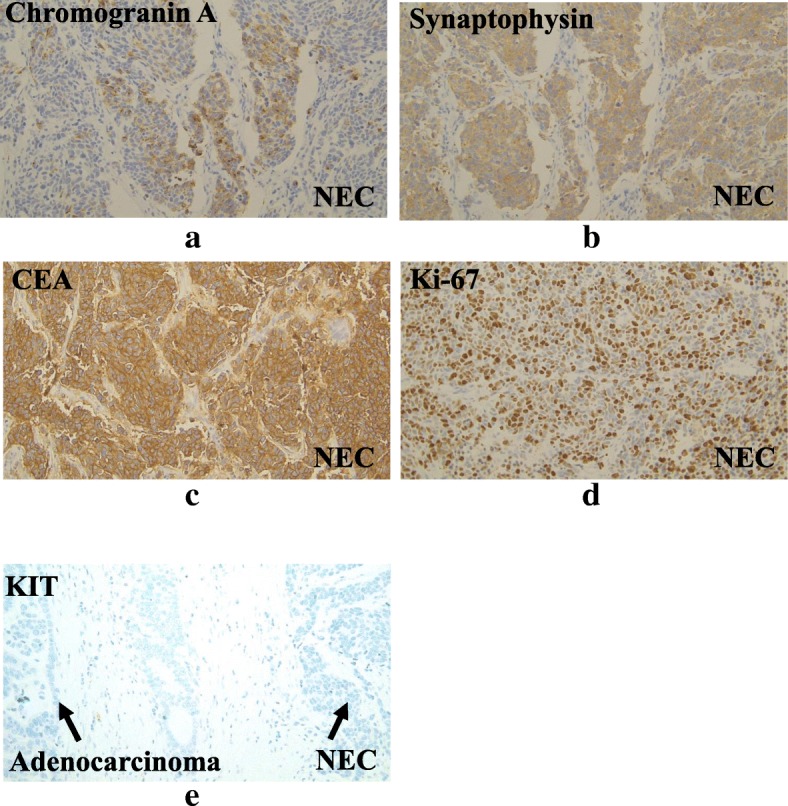
Immunohistochemical staining of the NEC revealed positive staining for chromogranin A (**a**), synaptophysin (**b**), and CEA (**c**), and more than 90% of the nuclei were positive for Ki-67 (**d**). Neither the adenocarcinoma component, nor the NEC component showed positive staining for KIT (**e**)

An 81-year-old man was referred to our hospital with a 5-month history of dysphagia. His medical history included diabetes mellitus and hypertension. Physical examination revealed no abnormalities. Laboratory examination revealed elevated blood levels of hemoglobin A1c (7.2%) and carcinoembryonic antigen (CEA) (9.6 ng/ml). A barium esophagogram revealed an ulcerative mass measuring 85 mm in length between the lower thoracic esophagus and abdominal esophagus (Fig. 1). Esophagogastroduodenoscopy (EGD) revealed an ulcerated mass involving the entire esophageal circumference in the lower thoracic esophagus, extending up to the esophagogastric junction (33 to 40 cm from the incisors) (Fig. 2). Histopathological examination of an endoscopic biopsy specimen showed proliferation of atypical cells with glandular structures, based on which the tumor was diagnosed as adenocarcinoma (Fig. 3). Computed tomography (CT) revealed wall thickening from the lower thoracic esophagus to the abdominal esophagus, but no evidence of lymph node or distant metastasis (Fig. 4). Positron emission tomography (PET)-CT revealed accumulation of fluorine-18-2-fluoro-2-deoxy-D-glucose (FDG) in the lower thoracic esophagus, with a standardized uptake value of 8.8 (Fig. 5). Thus, the patient was diagnosed as having esophagogastric junctional cancer (Siewert type I, T3N0M0, stage IIA: TNM-UICC 7th) and treated by thoracoscopic esophagectomy in the prone position, with three-field lymph node dissection and gastric tube reconstruction via a posterior mediastinal approach. The resected specimen revealed an ulcerated mass measuring 10.0 × 7.5 cm in size (Fig. 6a, b). Histopathological examination of the specimen revealed two distinct components, namely, an adenocarcinoma component and a neuroendocrine carcinoma component, with no zone of transition between the two (Fig. 6c, d). The neuroendocrine carcinoma (NEC) component, with small round tumor cells with a high nuclear/cytoplasmic ratio, was seen at the center of the tumor, while the tumor periphery was composed of the well-differentiated adenocarcinoma component. The tumor fulfilled the diagnostic criterion of MANEC of each of the two components comprising at least 30% of the tumor area. Immunohistochemically, the neuroendocrine component showed positive staining for chromogranin A, synaptophysin, and CEA, and the Ki-67 was more than 70% (Fig. 7a–d). The adenocarcinoma component showed positive staining for CEA. Neither component showed positive staining for KIT (CD117), which is a stem cell marker (Fig. 7e). The neuroendocrine component involved all layers of the gastrointestinal wall, with lymphovascular invasion. There was no lymph node metastasis. Therefore, the final pathological diagnosis was MANEC of the esophagogastric junction (T3N0M0, stage IIA). The patient was discharged from the hospital 32 days after the surgery, without any complications. Because the patient was elderly, a decision was taken against postoperative adjuvant chemotherapy. Six months after the surgery, the patient presented with liver metastasis. Thereafter, the tumor became even more aggressive, and the patient died 8 months after the surgery.

## Discussion

There are several reports in the literature suggesting that neuroendocrine tumors (NETs) contain an adenocarcinoma component. Nishikura et al. reported that 70.6% of all gastric endocrine cell carcinomas contain an adenocarcinoma component [2]. Maru et al. evaluated 40 patients with esophageal NEC and reported the presence of an adenocarcinoma component in 15 patients [3]. In the WHO classification published in 2010, MANEC was defined as a tumor containing both exocrine and endocrine components, with each component exceeding 30% of the total tumor area. The two carcinoma components in MANECs are usually represented by adenocarcinoma and NEC [1]. Because the histopathological findings of the tumor in our case were consistent with the aforementioned diagnostic criteria, our patient was diagnosed as having MANEC.

MANEC is a rare disease, and the exact frequency remains unknown. A search, to the best of our ability, of the PubMed database using the search terms “mixed adenoneuroendocrine carcinoma” and “esophagogastric junction” yielded only three cases of MANEC of the esophagogastric junction, including the present case (Table 1) [4, 5]. None of the cases was diagnosed as having MANEC prior to resection.

**Table 1:** Cases of mixed adenoneuroendocrine carcinoma of the esophagogastric junction

No.	Author	Year	Age	Gender	Symptoms	Morphological findings of the endoscopy	Initially diagnosis of the biopsy	Pathological TNM stage	Treatment	Period (months)	Prognosis
1	Veits [4]	2013	68	Male	Unknown	Rapidly growing mass	Barrett’s carcinoma	T1bNxMx	Endoscopic submucosal dissection	Unknown	Unknown
2	Juanmartinena [5]	2017	57	Male	Nausea, vomiting, dysphagia, weight loss	Ulcerative and stenotic mass	Neuroendocrine carcinoma	T3N3M0 StageIIIB	Ivor-Lewis esophagectomy	Unknown	Unknown
3	Our case	2018	81	Male	Dysphagia	Ulcerated mass	Adenocarcinoma	T3N0M0 Stage IIIA	Thoracoscopic esophagectomy	8	Died

MANEC manifests with non-specific symptoms, endoscopic findings, and radiological findings. It is considered as being difficult to diagnose prior to resection, is most often diagnosed by histopathological and immunohistochemical examination of the resected specimen. MANECs are exceedingly rare because of which no therapeutic strategies have been established yet. However, according to the WHO, surgery is the treatment of choice [1, 4]. In our case, the epicenter of the tumor was in the lower thoracic esophagus, and the tumor was classified as Siewert type I; therefore, the patient was treated by thoracoscopic esophagectomy with three-field lymph node dissection, to minimize the risk of mediastinal and cervical lymph node recurrence [6]. At our institute, thoracoscopic esophagectomy in the prone position is first applied for thoracic esophageal cancer. Thoracoscopic esophagectomy in the prone position has several advantages, including a wide surgical space generated by the effects of gravity, blood pooling outside the operative field, and a reduction in the risk of lung injury because of the absence of need to provide direct lung retraction [7]. It would seem to be a safe approach, especially for reducing the risk of pulmonary complications in elderly patients, like in our case.

Regarding chemotherapy, while the WHO classification of MANEC does not contemplate subcategories with assessment of the malignancy grade of each of the components, Lee et al. reported that the target of treatment in cases of MANEC should primarily be the more aggressive tumor component [8]. In other words, in cases of MANEC with a well-differentiated neuroendocrine component with a benign or low grade malignant behavior (NET-G1 or G2), chemotherapy should be focused on the more aggressive exocrine component, namely, the adenocarcinoma. In contrast, in those with NEC, the NEC would be the main target of therapy. Because MANECs usually consist of NEC and adenocarcinoma components [1], the chemotherapy would primarily consist of the regimens recommended for NEC. Thus, in the treatment of MANECs, it is necessary to carefully consider the pathological features in each individual case.

Although the carcinogenetic pathway of MANECs has not yet been clarified, two hypotheses have been proposed [9]. The first is that the originally present malignant exocrine cell is dedifferentiated, so that it develops into a neuroendocrine tumor. The other is that monoclonal multi-potent stem cells differentiate into two components. In our case, immunohistochemically, both the NEC and adenocarcinoma components showed positive staining for CEA, suggesting that the carcinogenetic pathway that was originally directed towards the development of adenocarcinoma switched to development of neuroendocrine carcinoma as a result of dedifferentiation of the malignant cells. Mondolf et al., in their case report of MANEC of the gallbladder, concluded, based on detailed immunochemical analysis of their case, that the profile was strongly suggestive stem cell tumor of the gallbladder [10]. In their case report, the tumor showed positive staining for KIT, a stem cell marker, and the tumor was considered to be of stem cell origin on the ground that the transitional tumor cells of both histological components showed positive staining for KIT. In our case, because both the malignant components were negative for KIT, the tumor may not have been derived from stem cells. Meanwhile, pathomorphologically, it has also been hypothesized that composite tumors with a transitional zone between two tumor components are derived from multidirectionally differentiated single neoplasms, whereas collision tumors without a transitional zone between two components are derived from multi-potent stem cells [11]. Because our patient was diagnosed as having a collision tumor with no transitional zone between the two tumor components, it is possible that the tumor was derived from stem cells. These findings are of great interest, not only in relation to the development of MANEC but also in relation to the development of NEC, and further research is needed.

## Conclusions

We have reported a patient with MANEC of the esophagogastric junction. Close attention should be paid to patients with MANEC, as this tumor can be highly aggressive and show rapid progression. In the treatment of MANEC, it is necessary to carefully consider the pathological features of each of the tumor components in each individual case.

## Abbreviations

MANEC: Mixed adenoneuroendocrine carcinoma
CEA: Carcinoembryonic antigen
EGD: Esophagogastroduodenoscopy
CT: Computed tomography
PET: Positron emission tomography
FDG: Fluorine-18-2-fluoro-2-deoxy-D-glucose
UICC: Union for International Cancer Control
NEC: Neuroendocrine carcinoma
NET: Neuroendocrine tumor
